# One-year mortality in patients requiring prolonged mechanical ventilation: multicenter evaluation of the ProVent score

**DOI:** 10.1186/cc13994

**Published:** 2014-07-18

**Authors:** Guillaume Leroy, Patrick Devos, Fabien Lambiotte, Didier Thévenin, Olivier Leroy

**Affiliations:** 1 Service de Réanimation Médicale et Maladies Infectieuses, Centre Hospitalier de Tourcoing, 135 avenue du Président Coty, 59208 Tourcoing, France; 2 Département de Biostatistiques, CHRU de Lille, 2 avenue Oscar Lambret, 59037 Lille, France; 3 Service de réanimation, Centre Hospitalier de Maubeuge, 13 boulevard Pasteur, 59600 Maubeuge, France; 4 Service de réanimation, Centre Hospitalier de Lens, 99 route de la Bassée, 62307 Lens, France

## Abstract

**Introduction:**

Current severity-of-illness indexes are unable to assess the long-term prognosis of patients requiring prolonged mechanical ventilation. A prognostic scoring system (Prognosis for Prolonged Ventilation score - ProVent - score) seems able to evaluate one-year mortality of such patients. However, testing of the model outside the developers' centers has not been reported. So, it is unclear how the ProVent score performs in non-US and non-tertiary ICUs. The goal of our study was to evaluate its performances in a French multicenter, community hospital-based setting.

**Methods:**

In three primary ICUs, 201 patients requiring mechanical ventilation for at least 21 days were enrolled in a retrospective cohort study. ICU mortality was abstracted from medical records and, for patients discharged alive from the ICU, one-year mortality was determined by telephone calls to patients’ general practitioners.

**Results:**

One-year mortality was 60% (n = 120). On day 21 of ventilation, ProVent score value was 0 in 19 patients (9%), 1 in 63 patients (31%), 2 in 64 patients (32%), 3 in 37 patients (18%), and ≥4 in 18 patients (9%), respectively. For ProVent score values ranging from 0 to ≥4, one-year mortality rates were 21%, 43%, 67%, 78%, and 94%, respectively. The area under the curve (AUC) of the receiver operator characteristic (ROC) curve for the ProVent score was 0.74 (95% confidence interval 0.671 to 0.809). Stepwise logistic regression analysis showed that only three variables (age ≥65 years, vasopressors, and hemodialysis) were independently associated with one-year mortality in our population. In assigning one point to each variable, we created a French ProVent score. The Hosmer-Lemeshow goodness-of-fit statistic was 1.36 (DF = 6, *P* = 0.857) and the AUC of the ROC curve was 0.742 (95% confidence interval 0.673 to 0.810). One-year mortality rates for French ProVent score ranging from 0 to 3 were 34.6%, 70.9%, 83.3% and 100%, respectively (*P* <0.0001).

**Conclusions:**

The ProVent score is able, even in non-US ICUs and in community hospitals, to accurately identify among patients requiring prolonged mechanical ventilation those who are at high risk of one-year mortality. Its simplification appears possible. However, further validation of this French ProVent score in a larger external sample is indicated.

## Introduction

Mechanical ventilation is the most frequent invasive technique applied to patients admitted to the intensive care unit (ICU). In the Extended Prevalence of Infection in Intensive Care (EPIC II) study, including 14,414 patients in 1,265 participating ICUs from 75 countries, more than half of the patients required acute mechanical ventilation during their ICU stay [1]. Among these mechanically ventilated patients, the National Association for Medical Direction of Respiratory Care estimated that 3 to 7% require prolonged mechanical ventilation defined as at least 21 days [2].

Such patients pose numerous problems. The consumption of a high amount of health-care resources, the high ICU and long-term mortality rates, and the difficulty to assess long-term prognosis must be underlined. Concerning this later point, Carson and Bach demonstrated in 2001 the inability of current severity-of-illness indexes such as acute physiology and chronic health evaluation score II, logistic organ dysfunction score, mortality prediction model II, and simplified acute physiology score II (SAPS II) to predict mortality of patients suffering from prolonged critical illness [3]. Conversely, Carson *et al*. demonstrated that a prognostic scoring system (Prognosis for Prolonged Ventilation – ProVent - score) was able to evaluate one-year mortality of patients requiring prolonged mechanical ventilation after initial intubation in tertiary care medical centers in the United States (US) [4,5]. However, it is unclear how the ProVent score performs in non-US and non-tertiary ICUs since, to the best of our knowledge, testing of the model outside the developers' centers has not been reported.

The goal of our study was to evaluate, in three French community hospitals, the performances of the ProVent score in a cohort of patients requiring prolonged mechanical ventilation in the ICU.

## Material and methods

### Patients

Patients were retrospectively enrolled between 1 January 2009 and 31 December 2011 from three primary ICUs of community hospitals (Lens, Maubeuge, and Tourcoing) in Nord-Pas de Calais, an area in the North of France. Lens, Maubeuge and Tourcoing hospitals were 1091-bed, 390-bed and 456-bed primary care medico-surgical centers with 16, 8 and 16 adult intensive care units beds, respectively. Institutional ethics approval was obtained from the Ethics Committee of Tourcoing Hospital (N°2013-01) as the responsible ethics committee. The Lens and Maubeuge institutional review boards were contacted and gave ethical approval for study participation. In accordance with French law, the need for informed patient consent was waived in our study because it is an observational retrospective cohort study that did not modify existing diagnosis or therapeutic strategies.

Inclusion criterion was duration of mechanical ventilation, after initial intubation, for at least 21 days during ICU stay. Exclusion criteria were age <18 years old, acute or chronic neuromuscular diseases, chronic diseases requiring invasive mechanical ventilation before ICU admission, and lack of data about patient characteristics or outcome.

### Data collection

In each site, cases were identified using a medical database query on the duration of mechanical ventilation. Data were retrospectively abstracted from medical records by the principal investigator at each site (DT, FL, and OL) and one abstractor who was blinded to patient outcome (GL).

Variables collected on ICU admission were age, gender, comorbidities, premorbid functional status (Knaus chronic health status score), admission diagnoses, and severity of illness (SAPS II, and sequential organ failure assessment (SOFA)) [6-8]. Chronic cardiac diseases included coronary heart diseases and heart failure (New York Heart Association class III to IV). Chronic obstructive pulmonary diseases were defined according to criteria proposed by the American Thoracic Society [9]. Chronic renal insufficiency was defined as a glomerular filtration rate <60 mL/min/1.73 m^2^[10]. Immunosuppression was defined by administration of steroid treatment in the six months prior to ICU admission (at least 0.3 mg/kg per day of a prednisolone equivalent for at least one month), and chemotherapy and/or radiotherapy in the six months prior to ICU admission.

On day 21 of mechanical ventilation, platelet count, and requirement of renal replacement therapy (on or within 48 hours of this day), and vasopressors were collected. In hospital outcome variables such as duration of mechanical ventilation, weaning from mechanical ventilation, tracheostomy during ICU stay, ICU length of stay, decision to withdraw and/or withhold life support, and ICU mortality were assessed. Finally, for patients discharged alive from ICU, one-year mortality was evaluated 12 months after day 21 of mechanical ventilation, and was determined by telephone calls to patients’ general practitioners.

ProVent score was determined for each patient as it was described by Carson *et al*. [5]. Briefly, this score is based on five predictor variables collected on day 21 of mechanical ventilation. Predictors and their point scoring system are age 50 to 64 years (+1 point), age ≥65 yrs (+2 points), platelet count <150 × 10^9^/L (+1 point), use of vasopressors (+1 point), and requirement for renal replacement therapy (+1 point). In adding the points, we obtain the ProVent score.

### Statistical analysis

Descriptive analyses were performed to check and resume data. Quantitative variables are reported as means ± standard deviation (SD). Qualitative variables are reported as number and percentage.

First, ProVent score was computed using point scoring derived from the β values provided by Carson *et al*. [5] and previously reported as follows: age 50 to 64 years (+1 point), age ≥65 yrs (+2 points), platelet count <150 × 10^9^/L (+1 point), use of vasopressors (+1 point), and requirement for renal replacement therapy (+1 point).

Second, we included the five predictor variables in a logistic regression model and assigned points to each predictor variable according to the β coefficients found in the model applied to our population. The Hosmer-Lemeshow goodness-of-fit statistic was used to assess the model.

Third, a stepwise logistic regression was used to select the best subset of independent variables and compute a French ProVent score. The Hosmer-Lemeshow goodness-of-fit statistic was also used to assess the model.

Predictive capabilities of the ProVent score using the point scoring as initially described by Carson *et al*., the ProVent score using the point scoring derived from our population, and, finally, the French ProVent score were compared using receiver operator characteristic (ROC) curves and area under the curve (AUC).

One-year survival curves were computed using the Kaplan-Meier estimates. Comparisons of survival curves according to the ProVent score were performed using the log-rank test.

Data were analyzed using the SAS software V9.3 (SAS Institute Inc., Cary, NC, USA).

## Results

A total of 246 medical records of patients requiring prolonged mechanical ventilation were studied. According to exclusion criteria (acute or chronic neuromuscular diseases n = 4, chronic diseases requiring invasive mechanical ventilation before ICU admission n = 7, and lack of data about patient characteristics n = 17 or outcomes n = 17), 45 patients were not enrolled.

Characteristics and outcomes of the remaining 201 patients are summarized in Table 1. Underlying comorbidities were present in 179 patients (89%). Two-thirds of patients were in class B or C of Knaus chronic health status score. Most patients (n = 150; 74%) exhibited a medical ICU admission diagnosis, mainly due to a cardiovascular or pulmonary disease.

**Table 1:** Patient characteristics and outcome

**Patient characteristics**	**n = 201**
Age, mean ± SD, years	64 ± 14
Male, n (%)	136 (68%)
Comorbidities, n (%)	
Chronic cardiac diseases	122 (61%)
Chronic respiratory diseases	78 (39%)
Chronic endocrine diseases	59 (29%)
Chronic hepatic diseases	31 (15%)
Chronic neurologic diseases	33 (16%)
Chronic renal insufficiency	14 (7%)
Immunosuppression	9 (4%)
Malignancy	21 (10%)
Human immunodeficiency virus infection	5 (2.5%)
Knaus chronic health status score	
Class A	32 (16%)
Class B	73 (36%)
Class C	64 (32%)
Class D	18 (9%)
Nonassessable	14 (7%)
ICU admission diagnoses, n (%)	
Cardiovascular including septic shock	68 (34%)
Surgery	46 (23%)
Pulmonary including pneumonia	43 (21%)
Neurologic	19 (9%)
Infection	10 (5%)
Gastrointestinal	6 (3%)
Endocrine	4 (2%)
Traumatic	5 (2%)
Hematologic or malignancy	0 (0%)
SAPS II ICU admission, mean ± SD	51 ± 17
SOFA ICU admission, mean ± SD	9 ± 4
Hospital outcomes	
Duration of MV, mean ± SD, days	37 ± 20
Duration of MV if died in ICU, mean ± SD, days	23 ± 29
Weaning from MV, n (%)	104 (52%)
Tracheostomy during ICU stay, n (%)	61 (30%)
ICU length of stay, mean ± SD, days	41 ± 21
Death in ICU, n (%)	83 (41%)
One-year mortality, n (%)	120 (60%)

SD, standard deviation; MV, mechanical ventilation; ICU, intensive care unit; SAPS, simplified acute physiology score; SOFA, sequential organ failure assessment.

Mean duration of mechanical ventilation was 37 ± 20 days. Liberation from mechanical ventilation was not obtained for 14 patients who were discharged from the ICU. Eighty-three (41%) patients died in the ICU. A decision to withdraw and/or withhold life support was made for 71 of 83 patients who died in the ICU. Among the 118 patients discharged alive from the ICU, 37 died during the year following discharge. So, one-year mortality was 60% (n = 120). The mean duration of survival following ICU discharge was 190 ± 289 days.

On day 21 of mechanical ventilation, 76 (37.8%) patients were 50 to 64 years old, 88 (43.8%) were ≥65 years old, 44 (21.9%) exhibited a platelet count <150 × 10^9^/L, 43 (21.4%) required vasopressors, and 40 (20%) required renal replacement therapy.

The number of patients included in the five groups (0 to ≥4) of the ProVent score and the one-year mortality rates for score values ranging from 0 to ≥4, in our study and in the second Carson’s study [5], were reported in Table 2. One-year mortality rates for ProVent score values ranging from 0 to ≥4, in our study and in Carson’s study, were not statistically different.

**Table 2:** **ProVent score and observed one-year mortality in Carson’s study**[5]**and our series**

**ProVent score**	**Carson’s study (5)**		**Our series**		
	**No.**	**Observed mortality percent (95% confidence interval)**	**No.**	**Observed mortality percent (95% confidence interval)**	***P*** *
0	72	20 (10-29)	19	21 (6-46)	0.9087
1	60	36 (24-48)	63	43 (30-56)	0.2568
2	78	56 (45-68)	64	67 (54-78)	0.0714
3	36	81 (67-94)	37	78 (62-90)	0.6844
4 or 5	14	100 (77-100)	18	94 (73-100)	0.0521

*Comparison between observed one-year mortality rates.

**Figure 1:**
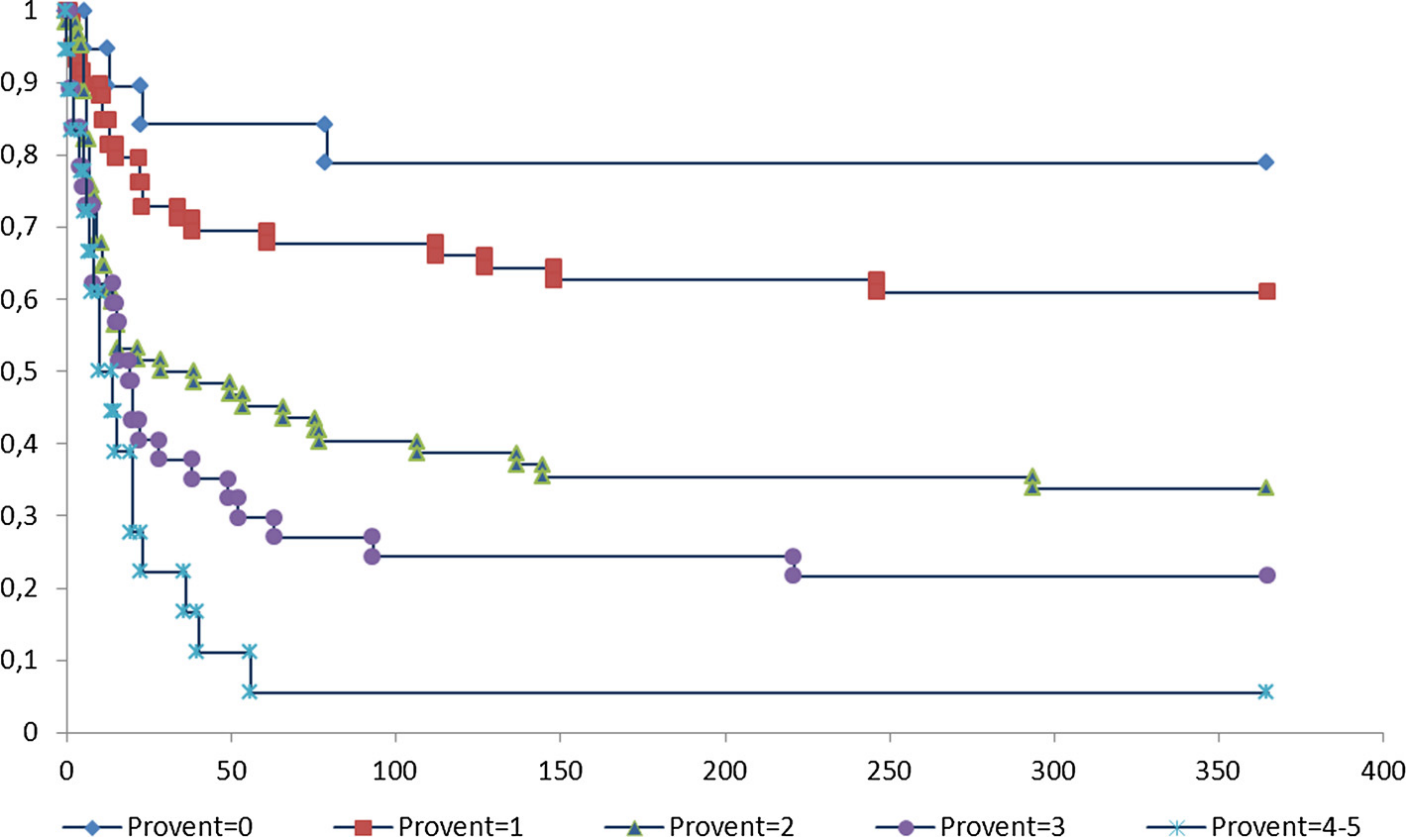
**Kaplan-Meier curve of one-year survival for patients by ProVent score.** X axis: number of days after day 21 of mechanical ventilation. Y axis: percentage of survival.

One-year survival curves, according to the values of ProVent score, are showed in Figure 1. Survival curves were statistically different (log-rank test: *P* <0.0001).

The results of the logistic regression model applied to our population are reported in Table 3. The analysis of this model suggests that, in our population, age 50 to 64 years and platelets ≤150 × 10^9^/L were not independently associated with one-year mortality and that, according to the β values observed in our population, a point scoring different from the point scoring reported by Carson *et al.*, could be proposed as follows: age ≥65 years = 3 points; platelets ≤150 x 10^9^/L = 1 point; vasopressors = 2 points and hemodialysis = 2 points. The Hosmer-Lemeshow goodness-of-fit statistic was 2.31 (DF = 6, *P* = 0.889).

**Table 3:** Model with five risk variables

**Categorical variable**	**Odds ratio**	**95% Confidence interval**	**β Value**	***P***
Age ≥65 years	4.495	1.872-10.791	1.5029	0.0008
Age 50-64 years	1.134	0.488-2.638	0.1259	0.7700
Platelets ≤150 × 10^9^/L	1.650	0.731-3.725	0.5007	0.2282
Vasopressors	3.326	1.355-8.163	1.2018	0.0087
Hemodialysis	3.410	1.278-9.102	1.2268	0.0143

Stepwise logistic regression analysis showed that only three variables (age ≥65 yrs., vasopressors, and hemodialysis) were independently associated with one-year mortality (Table 4). A final model was computed using only those three significant variables (Table 4). According to the β values observed in this model, one point was assigned to each variable to generate a French ProVent score. The Hosmer-Lemeshow goodness-of-fit statistic was 1.36 (DF = 6, *P* = 0.857). On day 21 of mechanical ventilation, the number of patients with a French ProVent score equal to 0, 1, 2 and 3 was 81, 79, 30 and 11, respectively. One year mortality rates for French ProVent score ranging from 0 to 3 were 34.6%, 70.9%, 83.3% and 100%, respectively (*P* <0.0001).

**Table 4:** Model with three variables

**Categorical variable**	**Odds ratio**	**95% Confidence interval**	**β Value**	***P***
Age ≥65 yrs	4.179	2.164-8.072	1.4302	<0.0001
Vasopressors	3.443	1.416-8.374	1.2365	0.0064
Hemodialysis	3.475	1.307-9.242	1.2456	0.0126

The AUC of the ROC curves for the ProVent score using point scoring reported by Carson *et al*., and point scoring proposed by our analysis are 0.74 (95% confidence interval 0.671 to 0.809) and 0.749 (95% confidence interval 0.680 to 0.817), respectively. The French ProVent score has an AUC of the ROC curve of 0.742 (95% confidence interval 0.673 to 0.810).

## Discussion

The main results of this multicenter study provide validation of the ProVent score in a country other than the USA and in primary ICUs of community hospitals. Furthermore, our data suggest that this score could be simplified.

Carson *et al*. demonstrated that four variables collected in day 21 of mechanical ventilation were independent predictors of one-year mortality of patients requiring prolonged mechanical ventilation [4]. These variables were age ≥50 years, requirement for vasopressors, hemodialysis, platelet count ≤150 × 10^9^/L. In a second study, they built a prognostic scoring rule based on the same risk variables but with two cut points for age. A point value was assigned to each of the five risk variables. The ProVent score was obtained by adding points [5]. In Carson *et al*. series [5] and in ours, one-year mortality rates were 48% and 60%, respectively. Comparison of mortality rates for ProVent score values ranging from 0 to ≥4, in these two studies, demonstrated no significant difference. The AUC of the ROC curve for the ProVent score was 0.74 in our series and 0.76 in the Carson *et al*. study [5]. Such data suggest that the ProVent score performs well in our series in identifying patients at lowest and highest risk of one-year mortality and, thus, that ProVent score could be used in countries other than the USA and in primary ICUs of community hospitals. Even if our results could suggest that a point scoring different from the initial point scoring reported by Carson *et al*. could be proposed, the values of the respective AUC of the ROC curves are quite similar, emphasizing the robustness of the ProVent model.

The results of the stepwise logistic regression analysis showed that in our series only three variables (age ≥65 yrs., vasopressors, and hemodialysis) were independently associated with one-year mortality. Two risk variables, age 50 to 64 years and platelet count ≤150 × 10^9^/L, were not significantly associated with one-year mortality. In our opinion, such a result is not surprising. In fact, in the Carson *et al*. study, the results of the logistic regression model already showed that the independent prognostic value of age 50 to 64 years and platelet count ≤150 × 10^9^/L could be questionable since odds ratios and 95% confidence intervals were 2.0 (1.0 to 3.9) and 1.9 (0.9 to 3.9), respectively [5]. In our study, in assigning one point to each risk variable we created a French ProVent score able to stratify the one-year prognostic of patients on day 21 of mechanical ventilation. Patients with a French ProVent score equal to 0, 1, 2 and 3 exhibited a significant increasing one-year mortality rate (34.6%, 70.9%, 83.3% and 100%, respectively). Of course, further validation of this French ProVent score in a larger external sample is indicated.

Long-term prognostication of patients requiring prolonged mechanical ventilation is challenging. Current severity-of-illness indexes are unable to predict mortality of patients suffering from prolonged critical illness [3]. Conversely, ProVent score appears as a simple, impartial and reproducible score able to accurately identify among patients requiring prolonged mechanical ventilation those who are at low or, conversely, at high risk of one-year mortality. However, the impact of the ProVent score on patient care and information given to patients, families, and surrogate decision makers remains to be determined. In our study, as in study of Carson *et al*., the score was not used for decisions of withholding or withdrawing life-sustaining treatment and communication with families. In our series, the decision to withdraw or withhold life support was made for 86% of patients who died in the ICU, but the ProVent score was never calculated before this decision. The two most important factors influencing decisions to withdraw or withhold life support are patient preferences and patient prognosis [11,12]. The use of a simple and reproducible score such as the ProVent score to give physicians reliable prognostic information could be interesting. Nevertheless, we must bear in mind the two following points: most often, physicians do not take into account prognostic information given by prognostic models, as demonstrated by the SUPPORT study [13]. Second, the translation of data from population-level outcomes to individual risk estimation has inherent limitations. Consequently, according to current ethical standards, the use of scoring systems as a unique tool to guide decisions of withdrawing or withholding life support is inappropriate [14].

Our study has several limitations and weaknesses. Briefly, it was a retrospective study. This point explains that 15% of studied patients were not enrolled in the cohort and that long-term functional status was not assessed. For the same reason, the impact of the ProVent score values on patient’s care and information given to patients, families, and surrogate decision makers was not studied. This later point is a major drawback of our study but represents a goal for future studies. Paired with clinical judgment, original or French ProVent scores should be evaluated, in futures studies, as a piece of the decision support process acceptable for physicians, patients and their family.

## Conclusions

The ProVent score could be used in countries other than the US and in primary ICUs of community hospitals. It appears as a simple, impartial and reproducible score able to accurately identify among patients requiring prolonged mechanical ventilation those who are at high risk of one-year mortality. Its simplification appears possible. However, further validation of this French ProVent score in a larger external sample is indicated. Moreover, the impact of these scores on patient care and information given to patients and family remains to be studied.

## Key messages

• The ProVent score is able to evaluate one-year mortality of patients requiring prolonged mechanical ventilation after initial intubation.

• It can be used in countries other than the US and in primary ICUs of community hospitals.

• A French ProVent score based on three predictors (age ≥65 years, vasopressors, and hemodialysis) performs as well as the original ProVent score.

• The impact of these scores on patient care and information given to patients and family remains to be studied.

## Abbreviations

AUC: area under the curve; ICU: intensive care unit; MV: mechanical ventilation; ROC: receiver operator characteristic; SAPS: simplified acute physiology score; SD: standard deviation; SOFA: sequential organ failure assessment; US: United States.

## Competing interests

All the authors declare that they have no competing interests.

## Authors' contributions

GL contributed to the design of the study, collected data and helped to draft the manuscript. PD performed the statistical analysis. FL collected data and helped to draft the manuscript. DT collected data and helped to draft the manuscript. OL contributed to the design of the study, collected data and wrote the manuscript. All authors read and approved the final manuscript.
